# Psychometric assessment of the 10-item Thai version of the Experience in Close Relationship-Revised for Adolescents (ECR-R-10-AD)

**DOI:** 10.1038/s41598-024-64437-2

**Published:** 2024-06-11

**Authors:** Tinakon Wongpakaran, Justin DeMaranville, Nahathai Wongpakaran, Danny Wedding

**Affiliations:** 1 Department of Psychiatry, Faculty of Medicine, Chiang Mai University, Chiang Mai, Thailand; 2 Multidisciplinary and Interdisciplinary School, Chiang Mai University, Chiang Mai, 50200 Thailand; 3 Department of Clinical and Humanistic Psychology, Saybrook University, Pasadena, CA 91103 USA; 4 Department of Psychology, University of Missouri-Saint Louis, St. Louis, MO 63121 USA; 5 Psychotherapy and Personality Disorders Unit, Department of Psychiatry, Faculty of Medicine, Chiang Mai University, 110 Intawaroros Road, T. Sriphum, A. Muang, Chiang Mai, 50200 Thailand

**Keywords:** Attachment, Child, Adolescent, Validity, Reliability, Confirmatory factor analysis, Measurement, ECR-R, Psychology, Health care

## Abstract

The 18-item version of the Experiences in Close Relationships-revised (ECR-R-18) is a valid and reliable scale used among Thai adolescents. However, it revealed problematic items that impacted the scale’s internal consistency. The study aimed to achieve two objectives: (1) develop a new, shorter scale by retaining only highly loaded items equally between attachment anxiety and attachment avoidance, and (2) evaluate the psychometric properties of the shorter ECR-R version compared to the existing 18-item scale. Objective 1 was achieved through Study 1, involving 204 youths aged 16–18 years (64% female). All participants completed the 18-item ECR-R, and exploratory factor analysis was conducted to identify suitable items for the new ECR-R-AD. Objective 2 was fulfilled in Study 2, which included a total of 443 students in grades aged 15–18 years old (88% female) from Thai boarding schools in Northern Thailand. All participants completed both the 18-item ECR-R, and confirmatory factor analysis of both the existing 18-item and the new shorter scale was performed and compared. Additional measures including the Rosenberg Self-Esteem Scale, Perceived Stress Scale-10, and Relationship Questionnaire were completed alongside the ECR-R to assess convergent, discriminant, and criterion validity. The invariance test for the new ECR-R across genders was conducted using multigroup confirmatory factor analysis. For objective 1, Study 1 developed a new scale called "ECR-R-10-AD" with 10 items, comprising 5 for attachment anxiety and 5 for attachment avoidance. The McDonald’s omega values were 0.866 for avoidance and 0.823 for anxiety subscales. The corrected correlation between the ECR-R-18 and ECR-R-10-AD was significant. For objective 2, Study 2 found that the first-order two-factor solution model fit the data best for the ECR-R-10-AD. Convergent, discriminant, and criterion validity with other measurements and invariance tests based on sex were established for the ECR-R-AD. The ECR-R-10-AD provided sufficient psychometric properties among Thai adolescents. Factorial validity, convergent validity, and measurement invariance were established. As the ECR-R-10-AD is brief, it can be administered with less burden. Limitations and future research were discussed.

## Introduction

Attachment measurement in adolescents involves assessing the quality and nature of their emotional bonds and relationships with significant others, typically their parents or caregivers. Other attachment measurement tools for adolescents may include interviews, observations, and projective tests, which aim to explore attachment patterns and dynamics in greater depth. These measurements help researchers and clinicians understand how adolescents form and maintain emotional connections, which can have significant implications for their psychological well-being and social development. Understanding attachment patterns in adolescents can also provide valuable insights into how early relationships influence their future adult relationships and overall mental health. Despite its significance, attachment studies among adolescents in Thailand appear to be underexplored. There is a limited number of attachment measurements specifically developed for Thai adolescents.

Several assessments gauge attachment between parents and adolescents. Examples encompass the Inventory of Parent and Peer Attachment^1^, Attachment Q-Sort^2^, Parental Bonding Instrument^3^, Child Attachment Interview^4^, and Adolescent Attachment Questionnaire^5^. Additional details regarding the dimensions of the attachment measured are provided in the Supplementary File 1.

Brennan and colleagues proposed two dimensions underlying attachment categories, labeled as anxiety and avoidance. These dimensions reflect an individual's level of discomfort and fear of abandonment in close relationships (anxiety) and their tendency to avoid emotional closeness and intimacy with others (avoidance). Later, they produced the Experiences in Close Relationships measure (ECR), a 36-item instrument assessing the two relatively orthogonal constructs of attachment anxiety and avoidance^6^. A revised version of the Experiences in Close Relationships (ECR-R) was subsequently developed by Fraley, Waller, and Brennan to improve the measure’s sensitivity across the range of possible scores^7^. The Thai version of the 36-item ECR-R was initially developed with permission from Fraley^8^. Subsequently, an 18-item version in Thai was later developed^9^.

The ECR-R is used for both adolescents and adults. The questionnaire consists of items that adolescents rate on a scale to indicate the extent to which each statement applies to them. In contrast to adults, where attachment styles are often assessed through relationships with romantic partners, attachment in adolescents is commonly measured within the context of their relationships with parents.

It is widely recognized that the use of questionnaires represents a valuable method for assessing the construct of attachment. Specifically, the anxiety and avoidance subscales have demonstrated a high level of validity in measuring this construct. Attachment-related anxiety refers to an individual's fear of being rejected by others, which is associated with a need for excessive attention and a tendency to exhibit intense stress reactions when a partner is unavailable or indifferent. On the other hand, attachment-related avoidance involves a tendency to reject dependency and interpersonal proximity, accompanied by self-absorption and a reluctance to disclose personal information. Individuals who receive high scores on one or both of these subscales are typically considered to have an insecure attachment style, while those who score low on both anxiety and avoidance subscales are generally regarded as having a secure attachment style^10^.

According to Fraley, simultaneous analysis of the two attachment dimensions in a regression framework enables interpretation of the results according to Bartholomew's four attachment prototypes: secure (low anxiety and avoidance), preoccupied (high anxiety and low avoidance), fearful (high anxiety and avoidance), and dismissing (low anxiety and high avoidance)^23^. The ECR-R has been employed in several research studies to predict psychological problems. One study indicated that change in adolescents' attachment anxiety and avoidance may be an important mechanism of change in adolescents' dysfunctional thinking patterns^11^, whereas another study demonstrated significant associations between childhood trauma exposure and risk of bipolar disorder^12^. A significant correlation between anorexia and insecure attachment measured by the ECR-R was also demonstrated^13^.

To address the challenge of the original ECR-R length, shortened versions were developed, though these were mostly in adult populations in various languages and settings. The number of items ranged from 12 to 18^14–17^. Our previous research has demonstrated which items of the ECR-R were suitable for inclusion in the abbreviated version for adults, focusing on the context of romantic partnerships^18^. For adolescents, Brenning and colleagues adapted the ECR-R to assess adolescents’ attachment with respect to their relationship with their parents (referred to as the ECR-RC). They argued that creating an attachment measurement from the ECR-R has some advantages. For example, ECR-R explicitly distinguishes between anxiety and avoidance instead of providing an overall assessment of attachment insecurity. It has a clear and interpretable factor structure and scales with strong internal consistency. Finally, it can be used in longitudinal research examining development in attachment from middle childhood to late adolescence and adulthood^19^.

The ECR-RC is a questionnaire that comprises of 36 statements related to the children's mother or father. It uses a scale from 1 (not at all) to 7 (very much) to measure the level of attachment anxiety or avoidance. The adaptation process included simplifying the item wording, removal of double negatives, and adjusting the content to be more relevant for children and their relationship with their parents. The results of the study suggest that the ECR-RC instrument is a promising tool^19^.

Brenning and colleagues then developed a brief, 12-item version of the ECR-RC that had very similar psychometric properties to those of the 36-item version. Specifically, the brief ECR-RC showed high levels of internal consistency, a stable two-factor structure, and virtually identical associations with external variables^20^.

In line with Brenning’s study, the Thai version of adult 18-ECR-R has been used in both clinical and nonclinical samples, with ages ranging from 18 to 95 years to predict psychological problems such as loneliness, anxiety, depression, and suicidality^21–29^. Recently, the adolescent version of the 18-item ECR-R has been developed based on the adult version^30^.

In some cases, using a more concise version of the 18-item Thai version of the ECR-R questionnaire may be beneficial without sacrificing its construct validity. A prior study found certain items in the original questionnaire to be problematic, negatively impacting the scale's reliability and overall validity^9^. Based on this, researchers recommend removing these items and adopting a shorter questionnaire version. Furthermore, many surveys in large community-based, school, or primary healthcare settings favor abbreviated versions of the ECR-R, given that attachment is often a central focus alongside mediators and outcomes^11,13,31^. Therefore, the length of the ECR-R questionnaire can pose difficulties for adolescents who have to fill out multiple questionnaires, especially if the ECR-R is lengthy.

In addition to the benefits of having a succinct and validated version of the ECR-R customized for adolescents, it's imperative to acknowledge the profound impact of cultural differences on respondents' interpretations of specific items^32–35^, particularly noticeable in Asian cultures, where individuals tend to exhibit attachment anxiety^27,35,36^. Notably, there is a research gap in the lack of any ECR-R specifically crafted for adolescents within an Asian cultural context.

To fill this gap, this study aims to derive a condensed scale version with robust internal consistency and predictive capability. This research was conducted by two studies; study 1, exploratory factor analysis was conducted on the 18-item ECR-R to find the most appropriate items for the short ECR-R (Sample 1, N = 204). Subsequently, in Sample 2 (N = 423), reliability analyses and Confirmatory Factor Analyses (CFA) were conducted to assess the psychometric properties of both the ECR-R-18 and the newly shorter ECR-R. Additionally, construct and criterion validity were evaluated for the new scale. The relationships between the shorter and existing ECR-R-18 versions were analyzed, and convergent validity of the both versions of the ECR-R and other relevant measurements were conducted.

## Materials and methods

This study was a cross-sectional online survey involving two studies.

### Sample size calculation

Comrey suggested a minimum sample size of 200 for factor analysis^37^. However, considering Mundform’s suggestion, the level of communality, and the variables per factor, the calculated minimum sample size was 70, based on 2 factors and 9 variables per factor^38^. This study analyzed two samples of 204 and 443 participants, ensuring sufficient statistical power.

### Participants

#### Study 1

To reduce the length of the ECR-R-18 questionnaire, a study was conducted which included 204 high school students aged 15–17. The participants had a mean age of 16.05 years with a standard deviation of 0.97. Additionally, 64% of the participants were female. All of the participants completed the 18-item ECR-R questionnaire. The aim of the study 1 was to evaluate the efficacy of shortening the questionnaire without compromising its validity and reliability.

#### Study 2

In the second study, we enrolled a total of 443 high school students from Thai boarding schools in Northern Thailand for the confirmatory factor analysis test of the shortened version of ECR-R. Given COVID-19, convenient sampling was deemed appropriate and employed for this study. Students were invited to participate and provided consent before completing the questionnaire via Google Forms. The data were gathered through digitally administered questionnaires via the Internet or computer software. Participants responded to questions using electronic devices such as computers, tablets, or smartphones.

Prior to participation, we obtained informed consent from all students, However, in the case of a few students (5 out of 423), they mentioned that their parents were unavailable and requested the school director to sign the document on their behalf. While it can be confirmed that permission was obtained from all parents, it's important to note that only five parents communicated their consent verbally and requested the school director to handle the documentation process on their behalf. The study received ethical approval from the ethics committee at the Faculty of Medicine, Chiang Mai University.

### Measurements

#### Adaptation of ECR-R-18 for adolescents

In line with Brenning’s ECR-RC, in this adolescent version, the word “romantic partner” was replaced by “parents.” Instead of using a separate question for mother or father, the authors used the word “parents” for the respondent to make an overall impression on how he/she feels towards both parents rather than to father or mother specifically.

The following measurements were used along with the ECR-R in each study.

*Rosenberg Self-Esteem Scale (RSES)*, a 4-point Likert scale that measures participant’s self-reported self-esteem^39^. Each item is scored on a scale ranging from strongly agree to strongly disagree, with response options usually assigned numerical values (e.g., strongly agree = 4, agree = 3, disagree = 2, strongly disagree = 1). Higher scores indicate higher levels of self-esteem. The Thai RSES has good internal consistency and good construct and discriminant validity^40^. In this study, Cronbach’s alpha for the RSES was 0.82.

*Perceived Stress Scale-10 (PSS-10)*, an instrument that measures the degree to which life events are perceived as being stressful^41^. This is a 5-point Likert scale instrument. Each item on the PSS-10 is scored on a 5-point Likert scale, ranging from 0 (Never) to 4 (Very Often). Higher scores indicate higher levels of self-esteem. The Thai PSS-10 has good internal consistency and good construct and discriminant validity^42^. In this study, Cronbach’s alpha for the PSS-10 was 0.85.

*Relationship Questionnaire (RQ)*, The RQ was developed by Bartholomew and Horowitz (1991) and consists of four distinct categories of attachment styles: secure, fearful, preoccupied, and dismissing^43^. Unlike other measures, the RQ is a single-item measure that utilizes four short paragraphs. Each paragraph describes a typical attachment pattern as it pertains to close relationships in adulthood. The RQ questionnaire is divided into two parts: RQ1 and RQ2. In RQ1, participants are asked to select the paragraph that best describes them without providing a numerical rating. For example, one of the statements for secure attachment might read as follows: "It is easy for me to become emotionally close to others." In RQ2, participants are then asked to rate their agreement with each attachment prototype on a 7-point scale. The rating provided for the attachment prototype with the highest score is used to classify participants into their corresponding attachment categories. RQ provides insights into two dimensions: the model of the self and the model of the other. By employing the RQ, researchers can comprehensively understand individuals' attachment styles and perceptions of themselves and others within close relationships. RQ is a valid self-reported measurement as long as an agreement between RQ1 and RQ2 is established^23^.

### Statistical analyses

Descriptive statistics were used to screen the data, ensuring that all items exhibited skewness and kurtosis values within the range of ± 2, as recommended by Nunnally and Bernstein^44^. Missing values were handled by replacing them with the mean of the respective item series.

In study 1, an exploratory factor with oblique rotation methods was conducted. Parallel analysis was performed to ascertain the number of factors by comparing the eigenvalues obtained from the actual data with those obtained from randomly generated data. This process aids in determining the number of factors to retain in factor analysis, considering only those factors with eigenvalues greater than the corresponding eigenvalues from the random data. Items for the new brief scale were chosen based on their factor loading levels (> 0.4). We ran a parallel analysis and scree plot in JASP 0.18.3 (2024).

In study 2, confirmatory factor analysis was conducted in both the original 18-item and short versions based on study 1. Model fit was evaluated using various indices, including the Comparative Fit Index (CFI) ≥ 0.95, Tucker-Lewis Index (TLI) ≥ 0.9, root-mean-square error of approximation (RMSEA) ≤ 0.6, and standardized root-mean-square residual (SRMR) ≤ 0.08, following the guidelines of Hu and Bentler^45^. Convergent and discriminant validity were determined by calculating Pearson's correlation coefficients between the ECR-R and other measures. To account for spurious correlations when evaluating the short-form, the corrected correlation coefficients obtained through Levy's method were used to calculate the correlation coefficients between the ECR-R-18 and the short ECR-R^46^. Internal consistency reliability was evaluated using McDonald's omega coefficient, with a reliability value greater than 0.70 considered acceptable.

Criterion validity was assessed by examining the regression model's predictive coefficients (B) of anxiety and avoidance for each attachment style. Following the theoretical model and a recommendation by Fraley, the expected B values for each attachment style are as follows: Secure Attachment: The B values for anxiety and avoidance should be negative (-B) and negative (-B), respectively. Fearful Attachment: The B values for anxiety and avoidance should both be positive (+ B). Preoccupied Attachment: The B value for anxiety should be positive (+ B), while the B value for avoidance should be zero (0B). Dismissing Attachment: The B value for anxiety should be zero (0B), while the B value for avoidance should be positive (+ B). These analyses were conducted using IBM SPSS version 22 and AMOS package version 22.

Multigroup confirmatory factor analysis was performed separately for males and females to examine the invariance of the new ECR-R. Measurement invariance analyses were conducted based on the criteria proposed by Byrne^47^, with CFI and TLI ≥ 0.90, and RMSEA ≤ 0.1^48^. Regarding the Measurement Invariance (MI), the original data consisted of 390 females and 53 males, with a ratio of 7.4:1. To address the extreme imbalance, we created a new dataset comprising 159 participants, with a ratio of 2:1 (106 females and 53 males), achieved through case-matching based on age. While the sample size remains modest, alternative model fit statistics, such as the Comparative Fit Index (CFI), can be utilized as they are less influenced by sample size. The results were comparable to the original results using the whole sample (n = 443) analysis.

Goodness-of-fit statistics were estimated for each model and compared to the less restricted model. Following the recommendations outlined by Byrne, three models were tested. These models progressively constrained the number of items and factors (configural invariance), factor loadings (metric invariance), and thresholds (scalar invariance) to be equal across groups. Maximum Likelihood Robust (MLR) estimator was used for multigroup confirmatory factor analysis. The differences between the adjustments of the most restricted and least restricted models were evaluated using Δχ2 (difference in chi-square value), ΔCFI, ΔTLI and ΔRMSEA. The values greater than 0.01 for ΔCFI and ΔTLI, and values larger than 0.015 for ΔRMSEA indicated a significant decline in fit^48,49^. Mplus 8.10 version was used to perform these analyses.

### Ethics approval and consent to participate

The study was conducted according to the guidelines of the Declaration of Helsinki and approved by the Institutional Review Board (or Ethics Committee) of the Faculty of Medicine, Chiang Mai University (EC: PSY-2564-08146, approved date 10 June 2021).

## Results

### Study 1: Determining the suitable number of items for the new ECR-R

The exploratory factor analysis of the 18-item Thai ECR-R revealed that within the anxiety dimension, nine items exhibited factor loadings ranging from 0.462 to 0.755, while the avoidance dimension featured five items with factor loadings ranging from 0.333 to 0.856. The scree plot also suggested a two-factor solution model. In the parallel analysis, the first three eigenvalues from the raw data of the ECR-R-18 were 5.718, 2.589, and 1.163. Correspondingly, the first three 95th-percentile random-data eigenvalues were 1.361, 1.284, and 1.231, suggesting that two factors were the most suitable structure for the ECR-R-18 (refer to Supplementary Files 2). Considering loadings above 0.4, the avoidance dimension yielded five items with loadings ranging from 0.622 to 0.856. To ensure an even distribution within each dimension^50^, we selected five items for the anxiety dimension, with factor loadings ranging between 0.638 and 0.755. Consequently, there were five items for each dimension, totaling 10 items for the scale. This modified version of the ECR-R is denoted as ECR-R-10-AD henceforth (see Supplementary File 3).

### Study 2: Validity and reliability of the ECR-R-10-AD

The characteristics of the respondents were as follows, 88% female, mean age 16.35 [SD 0.96]). The number of participants in each grade level were almost equal. The family income was almost equal between the two groups. Most of the participants were taken care of by their parents (Table 1).

**Table 1:** Sociodemographic Characteristics of the Participants.

Variables	N (%) or Mean ± SD
Age	16.35 ± 0.96
Sex, female	390(88)
Class	
Grade 10	144(32.5)
Grade 11	158(35.7)
Grade 12	141(31.8)
Family income	
Less than USD 295 *	237(53.9)
296 and higher	206(46.1)
Caretaker	
Both parents	178(40.2)
Father or mother	117(26.4)
Parent with relatives	105(23.7)
Relatives only	43(9.7)

* 1 USD = 32 THB (exchange rate at time of study), SD = standard deviation.

Table 2 shows the mean and standard deviation of the perceived stress and Rosenberg self-esteem scores. The sample showed a low level of perceived stress, whereas a high level of self-esteem. Most participants reported having a secure attachment, and the type of insecurity was preoccupied.

**Table 2:** Descriptive statistics of psychosocial variables.

Variables	N (%) or Mean ± SD
Perceived stress scale	23.94 ± 5.43
Rosenberg self-esteem scale	20.57 ± 4.14
Relationship Questionnaire (n = 443)	
Secure style	239(54.0)
Fearful style	55(12.4)
Preoccupied style	91(20.5)
Dismissing style	58(13.1)

*SD* standard deviation.

Factor loading coefficients of each item and the correlation between latent scores of anxiety and avoidance subscale scores are presented in Table 3. The factor loading coefficients ranged from 0.631 to 0.797 for anxiety and 0.574 to 0.906 for avoidance. The data fit the first-order two-factor solution model well, as indicated by χ^2^ (df) = 94.380 (34), p < 0.001, CFI = 0.968, TLI = 0.957, RMSEA = 0.063 (0.048–0.079), and SRMR = 0.046.

**Table 3:** Factor loading coefficients of ECR-R-10 items.

	Estimate	S.E	Est./S.E	P-Value
Anxiety				
ECRR2	0.696	0.03	22.85	< 0.0001
ECRR4	0.631	0.034	18.582	< 0.0001
ECRR8	0.797	0.025	32.308	< 0.0001
ECRR14	0.641	0.034	19.104	< 0.0001
ECRR16	0.726	0.029	25.331	< 0.0001
Avoidance				
ECRR3	0.574	0.035	16.568	< 0.0001
ECRR5	0.586	0.034	17.287	< 0.0001
ECRR7	0.811	0.020	40.42	< 0.0001
ECRR11	0.819	0.019	42.689	< 0.0001
ECRR15	0.906	0.015	62.446	< 0.0001
Anxiety and avoidance	0.366	0.049	7.539	< 0.0001

*S.E.* standard error.

Table 4 presents the mean and standard deviation of the ECR-R. A strong correlation between the long and short scales was observed, with Levy’s corrected correlation coefficients ranging from 0.757 to 0.777 for the avoidance and anxiety subscales, respectively. No significant differences were found in the correlation coefficients and corrected correlation coefficients between ECR-R-18 and ECR-R-10. Regarding McDonald’s Omega, the value for ECR-R avoidance was 0.778. However, this value increased to 0.838 when item #1 (I prefer not to show my parents how I feel deep down) was removed.

**Table 4:** Mean and standard deviation of ECR-R-18 and ECR-R-10.

	Avoidance		Anxiety	
	ECR-R-18	ECR-R-10	ECR-R-18	ECR-R-10
Mean ± SD	2.97 ± 0.96	2.80 ± 1.00	2.72 ± 1.33	2.42 ± 1.17
Pearson’s correlation	0.904**		0.932**	
Levy’s Corrected correlation	0.757		0.777	
McDonald’s omega coefficient	0.778(0.838)^†^	0.868	0.861	0.825

*SD* standard deviation.

^†^Omega coefficient when item #1 was removed.

** p < .001.

Table 5 displays the correlation coefficients between other measurements and both the ECR-R-18 and ECR-R-10-AD. Discrepancies were identified in the correlation coefficients between the avoidance and anxiety subscales of ECR-R-18 and ECR-R-10-AD, as well as the RSES and PSS. However, no significant differences were observed in the correlation coefficients between ECR-R-18, RSES, and PSS, or between ECR-R-10-AD, RSES, and PSS (statistical results not displayed).

**Table 5:** Correlation coefficients between avoidance and anxiety subscales of ECR-R-18 and ECR-R-10.

Measurements	ECR-R-18		ECR-R-10	
	Avoidance	Anxiety	Avoidance	Anxiety
RSES	− 0.355**	− 0.523**	− 0.395**	− 0.453**
PSS	0.397**	0.278**	0.315**	0.323**

*RSES* Rosenberg self-esteem scale, *PSS-10* Perceived stress scale

Table 6 compares the unstandardized coefficients for anxiety and avoidance scores in predicting attachment styles. The ECR-R-10-AD demonstrates anticipated coefficients (B) compared to the ECR-R-18. For instance, in secure attachment, the coefficients were − 0.043 (p < 0.001) and − 0.183 (p = 0.090) for ECR-R-18, while for ECR-R-10-AD, they were − 0.255 (p < 0.001) and − 0.230 (p = 0.003), suggesting a theoretically sounder representation by ECR-R-10-AD. Unlike ECR-R-18, which could only confirm two types (i.e., Fearful and Preoccupied), ECR-R-10-AD provided relevant coefficients based on the respective attachment style as per the research question (RQ).

**Table 6:** Comparison of RQ Attachment style predicted by anxiety and avoidance dimension scores between ECR-R-18 and ECR-R-10.

ECR-R-18		B	S.E	Wald	df	p-value	Exp (B)
Secure	Anxiety	− 0.048	0.011	19.779	1	< 0.001	0.953
	Avoidance	− 0.183	0.108	2.874	1	0.090	0.832
	Constant	1.896	0.369	26.357	1	< 0.001	6.656
Fearful	Anxiety	0.053	0.015	13.340	1	< 0.001	1.055
	Avoidance	0.325	0.142	5.258	1	0.022	1.385
	Constant	− 4.304	0.566	57.861	1	< 0.001	0.014
Preoccupied	Anxiety	0.138	0.027	25.036	1	< 0.001	1.147
	Avoidance	− 0.294	0.279	1.108	1	0.293	0.745
	Constant	− 6.386	1.129	32.000	1	< 0.001	0.002
Dismissing	Anxiety	0.022	0.015	2.180	1	0.140	1.022
	Avoidance	0.262	0.142	3.396	1	0.065	1.300
	Constant	− 3.254	0.525	38.369	1	< 0.001	0.039
ECR-R-10							
Secure	Anxiety	− 0.255	0.087	8.509	1	0.004	0.775
	Avoidance	− 0.230	0.077	8.900	1	0.003	0.795
	Constant	1.424	0.281	25.607	1	< 0.001	4.155
Fearful	Anxiety	0.269	0.117	5.297	1	0.021	1.309
	Avoidance	0.304	0.102	8.788	1	0.003	1.355
	Constant	− 3.480	0.441	62.264	1	< 0.001	0.031
Preoccupied	Anxiety	1.105	0.219	25.575	1	< 0.001	3.020
	Avoidance	− 0.021	0.195	0.011	1	0.915	0.979
	Constant	− 6.545	0.992	43.543	1	< 0.001	0.001
Dismissing	Anxiety	0.146	0.120	1.474	1	0.225	1.157
	Avoidance	0.216	0.104	4.322	1	0.038	1.241
	Constant	− 2.902	0.420	47.765	1	< 0.001	0.055

Table 7 presents the fit measures of the multi-group models used to test measurement invariance across sexes. No significant differences in Δχ2 were observed between metric and configural, between scalar and metric, and between configural and scalar models (all p > 0.05). The data supported the scalar invariance model. In addition, the ΔCFI, ΔTLI, and ΔRMSEA were below the cutoff values suggesting that the measurement invariance between male and female was established.

**Table 7:** Model comparisons for measurement invariance testing across sex.

Model	χ^2^ (*df*)	Δχ^2^ (Δ*df*)	CFI	TLI	SRMR	RMSEA	ΔCFI	ΔTLI	ΔRMSEA
Configural	108.505 (68)		0.944	0.926	0.077	0.087			
Metric	115.029(76)	6.523(8), p = 0.588	0.946	0.936	0.083	0.080	0.002	0.010	0.007
Scalar	128.590(84)	13.562(8), p = .093	0.951	0.934	0.086	0.082	0.005	0.002	0.002

*χ*^*2*^ chi-squared test, *df* degrees of freedom, *CFI* comparative fit index, *TLI* Tucker--Lewis index, *SRMR* standardized root mean square residual, *RMSEA* root mean square error of approximation.

## Discussion

The abbreviated Thai version of the ECR-R-10-AD has shown significant improvement in its psychometric properties after excluding a poorly fitting item. The new version has surpassed the previous ECR-R-18 version and has the potential for wider application in relevant research and clinical settings.

The adolescent data have confirmed factorial validity by excellently fitting the two-factor solution model. Convergent validity with other measurements was established. In addition, the ECR-R-10-AD held better criterion validity against the relationship questionnaire than the ECR-R-10. Both versions demonstrated good internal consistency. Therefore, the ECR-R-10-AD is favorable because it has fewer items.

Consistent with other related studies, the ECR-R-10-AD demonstrated measure invariance across sexes, suggesting that the tool is measuring the same construct in a similar manner for both males and females^51,52^. The measurement's validity and reliability are not significantly influenced by gender differences, meaning that the scale's items are equally meaningful and relevant for both sexes. Any observed differences in the scores between sexes are not due to measurement bias or inconsistencies but rather reflect genuine differences in the construct being measured.

In line with the 12-item ECR-RC, the ECR-R-10-AD demonstrated relatively superior psychometric properties than the 18-ECR-R^20,53^. When comparing the items selected for the ECR-R-10-AD and the 12-item ECR-RC by Brenning and colleagues, it shows that 70% of the items of the ECR-R-10-AD were included in ECR-RC. However, the invalid item #1, “I prefer not to show my parents how I feel deep down,” of the ECR-R-10-AD, is found to be valid for the ECR-RC ( as well as the other short ECR-R)^54^. It is difficult to conclude whether the adolescent version of ECR-R is influenced by culture. The ECR-R-10-AD is developed from the ECR-R-18 tested in a young adult population, not from the 36-item ECR-R as in Brenning’s study. Despite that, over 70% are overlapping. Based on the previous study of cross-cultural attachment^35^, it might be possible that these two versions of the ECR-R-10-AD and the ECR-RC might be suitable for participants from different cultures (e.g., European and Asian).

Compared to the recent ECR-R-10 designed for adults^18^, it has been observed that they feature distinct sets of items. While all five items in the anxiety subscale remain consistent, there are differences in the avoidance subscale between adults and adolescents. In the adolescent version (ECR-R-10-AD), the items "I find it relatively easy to get close to my parents." and "I talk things over with my parents." are included, whereas these items are absent in the adult version (ECR-R-10). Instead, the adult version features items like "I find it easy to depend on romantic partners." and "It helps to turn to my romantic partner in times of need." These findings underscore the necessity for developing separate scales for these age groups, as the target relationships vary significantly.

### Clinical implications

Given its robust psychometric properties, the 10-item version is a viable and recommended alternative to the 18-item questionnaire. It can be used in adolescent attachment research.

### Limitations and future research

Despite the merits of brevity and wide use, the ECR-R-10-AD has some limitations. The sample used for analysis might not represent general adolescents because they were from boarding schools. As the sample size used for Measurement Invariance (MI) was small, there is a possibility of bias in the results. Therefore, interpretation should be approached with caution. Secondly, test–retest reliability has not been examined in all studies, and further investigation of this tool’s stability should be conducted. Finally, the fact that the adolescents’ attachment to their father or mother may be different, further investigation should be explored for different effects. Nonetheless, the current ECR-R-10-AD seems to be a promising brief instrument for measuring anxious and avoidant attachment representations in children and adolescents.

## Conclusion

The ECR-R-10-AD provided sufficient psychometric properties among adolescents. Factorial validity, convergent validity and measurement invariance were established. As the ECR-R-10-AD is brief, it can be administered with less burden. However, test–retest reliability and measurement invariance test should be further investigated and confirmed.

### Supplementary Information

Supplementary Information 1.

Supplementary Information 2.

Supplementary Information 3.

## Data Availability

The datasets used and/or analyzed during the current study are available from the corresponding author upon reasonable request.

## Abbreviations

CFA: Confirmatory factor analysis
CFI: Comparative Fit Index
ECR-R: Experience in Close Relationship-Revised
PSS: Perceived stress scale
RMSEA: Root Mean Square Error of Approximation
RQ: Relationship Questionnaire
RSES: Rosenberg Self-Esteem Scale
SRMR: Standardized root-mean-square residual
TLI: Tucker–Lewis Index
