# Supplementary material for: Psychometric assessment of the 10-item Thai version of the Experience in Close Relationship-Revised for Adolescents (ECR-R-10-AD)
Source: Sci Rep. 2024 Jun 11;14:13408. doi: 10.1038/s41598-024-64437-2 (PMC11166994; doi:10.1038/s41598-024-64437-2)
Supplement: Supplementary file 1 — Supplementary Information 1. [file 41598_2024_64437_MOESM1_ESM.docx]

| Measurements used in adolescents | | |
| --- | --- | --- |
| Questionnaires | Relationship assessed | Subscale / dimension |
| The Inventory of Parent and Peer Attachment (IPPA) | Parent- child and peer attachment | Trust, communication, and alienation in relationships with parents and peers. |
| The Attachment Q-Sort (AQS) | parent-child | Trust, emotional availability, and responsiveness |
| The Parental Bonding Instrument (PBI) | parent-child | The perceived levels of parental warmth, affection, and protection on one hand (Care), and the perceived levels of control, intrusion, and overprotection on the other hand (Overprotection). |
| The Child Attachment Interview (CAI) | parent-child | Emotional Openness, Balance of positive/negative references to attachment figures, Use of examples, Preoccupied Anger, Resolution of conflicts, Idealization, Dismissal, Atypical/Disorganized behavior, and Overall coherence. |
| The Adolescent Attachment Questionnaire (AAQ) | Parent- child and peer attachment | Cognitive, emotional, and behavioral aspects of attachment relationships |
