# Supplementary material for: Psychometric assessment of the 10-item Thai version of the Experience in Close Relationship-Revised for Adolescents (ECR-R-10-AD)
Source: Sci Rep. 2024 Jun 11;14:13408. doi: 10.1038/s41598-024-64437-2 (PMC11166994; doi:10.1038/s41598-024-64437-2)

| Factor Loadings coefficients of the ECR-R-18 | | | |
| --- | --- | --- | --- |
|  | Factor 1 | Factor 2 | Uniqueness |
| ECRR8 | **0.755** | -0.012 | 0.437 |
| ECRR16 | **0.752** | -0.047 | 0.459 |
| ECRR4 | **0.687** | -0.172 | 0.590 |
| ECRR14 | **0.683** | 0.029 | 0.517 |
| ECRR2 | **0.638** | 0.072 | 0.552 |
| ECRR12 | 0.589 | 0.032 | 0.637 |
| ECRR6 | 0.575 | 0.192 | 0.547 |
| ECRR18 | 0.530 | 0.152 | 0.634 |
| ECRR10 | 0.462 | 0.045 | 0.768 |
| ECRR13 | 0.165 | 0.333 | 0.819 |
| ECRR15 | 0.067 | **0.856** | 0.219 |
| ECRR7 | -0.023 | **0.799** | 0.376 |
| ECRR11 | 0.015 | **0.770** | 0.398 |
| ECRR5 | -0.047 | **0.657** | 0.590 |
| ECRR3 | -0.067 | **0.622** | 0.640 |
| ECRR9 | -0.037 | 0.451 | 0.809 |
| ECRR17 | 0.049 | 0.490 | 0.739 |
| Note. Applied rotation method is oblimin. | | | |

| **Parallel Analysis** | | | | | |
| --- | --- | --- | --- | --- | --- |
|  | | Real data component eigenvalues | | Simulated data mean eigenvalues | |
| Factor 1* |  | 5.718 |  | 1.361 |  |
| Factor 2* |  | 2.589 |  | 1.284 |  |
| Factor 3 |  | 1.163 |  | 1.231 |  |
| Factor 4 |  | 0.914 |  | 1.178 |  |
| Factor 5 |  | 0.867 |  | 1.135 |  |
| Factor 6 |  | 0.782 |  | 1.096 |  |
| Factor 7 |  | 0.692 |  | 1.060 |  |
| Factor 8 |  | 0.626 |  | 1.022 |  |
| Factor 9 |  | 0.591 |  | 0.986 |  |
| Factor 10 |  | 0.521 |  | 0.954 |  |
| Factor 11 |  | 0.472 |  | 0.917 |  |
| Factor 12 |  | 0.444 |  | 0.887 |  |
| Factor 13 |  | 0.430 |  | 0.855 |  |
| Factor 14 |  | 0.365 |  | 0.817 |  |
| Factor 15 |  | 0.331 |  | 0.780 |  |
| Factor 16 |  | 0.284 |  | 0.747 |  |
| Factor 17 |  | 0.209 |  | 0.690 |  |
|  | | | | | |
| *Note.*  '*' = Factor should be retained. Results from PC-based parallel analysis. | | | | | |
|  | | | | | |

### Scree plot


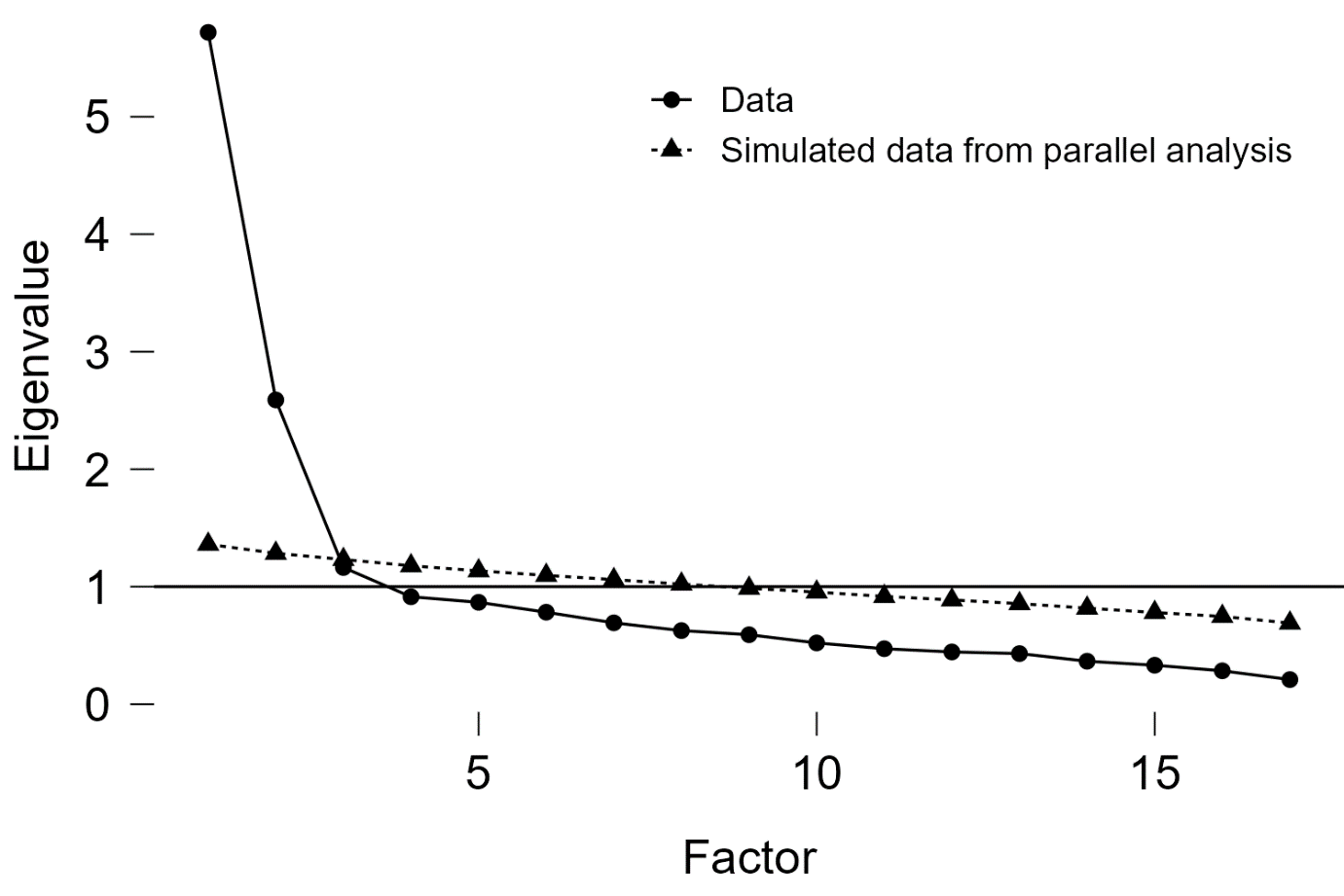

Supplement: Supplementary file 2 — Supplementary Information 2. [file 41598_2024_64437_MOESM2_ESM.docx]
