# Supplementary material for: Psychometric assessment of the 10-item Thai version of the Experience in Close Relationship-Revised for Adolescents (ECR-R-10-AD)
Source: Sci Rep. 2024 Jun 11;14:13408. doi: 10.1038/s41598-024-64437-2 (PMC11166994; doi:10.1038/s41598-024-64437-2)
Supplement: Supplementary file 3 — Supplementary Information 3. [file 41598_2024_64437_MOESM3_ESM.docx]

| **The items of the ECR-R-10-AD** |
| --- |
| *Anxiety* |
| 1. I often worry that my parents don’t really love me. |
| 2 When my parents are out of sight, I worry that they might become interested in someone else. |
| 3. I often worry that my parents will not want to stay with me. |
| 4. Sometimes parents change their feelings about me for no apparent reason. |
| 5. I worry that parents won’t care about me as much as I care about them. |
| *Avoidance* |
| 1. I feel comfortable sharing my private thoughts and feelings with my parents. |
| 2. I find it relatively easy to get close to my parents. |
| 3. I usually discuss my problems and concerns with my parents. |
| 4. I tell my parents just about everything. |
| 5. I talk things over with my parents. |
|  |
